# Antibiotic treatment triggers gut dysbiosis and modulates metabolism in a chicken model of gastro-intestinal infection

**DOI:** 10.1186/s12917-018-1761-0

**Published:** 2019-01-25

**Authors:** Caroline Ivanne Le Roy, Martin John Woodward, Richard John Ellis, Roberto Marcello La Ragione, Sandrine Paule Claus

**Affiliations:** 1 Department of Food and Nutritional Sciences, University of Reading, Whiteknights, Reading, RG6 6AP UK; 2 Present Address: Department of Twin Research & Genetic Epidemiology, King’s College London, London, SE1 7EH UK; 3 Central Sequencing Unit, Animal and Plant Health Agency, Addlestone, Surrey, KT15 3NB UK; 4 Faculty of Health and Medical Sciences, School of Veterinary Medicine, University of Surrey, Guilford, Surrey, GU2 7AL UK

**Keywords:** Microbiota, Metabolism, Antibiotic, Energy, Dysbiosis

## Abstract

**Background:**

Infection of the digestive track by gastro-intestinal pathogens results in the development of symptoms ranging from mild diarrhea to more severe clinical signs such as dysentery, severe dehydration and potentially death. Although, antibiotics are efficient to tackle infections, they also trigger dysbiosis that has been suggested to result in variation in weight gain in animal production systems.

**Results:**

Here is the first study demonstrating the metabolic impact of infection by a gastro-intestinal pathogen (*Brachyspira pilosicoli*) and its resolution by antibiotic treatment (tiamulin) on the host (chicken) systemic metabolism and gut microbiota composition using high-resolution ^1^H nuclear magnetic resonance (NMR) spectroscopy and 16S rDNA next generation sequencing (NGS). Clear systemic metabolic markers of infections such as glycerol and betaine were identified. Weight loss in untreated animals was in part explained by the observation of a modification of systemic host energy metabolism characterized by the utilization of glycerol as a glucose precursor. However, antibiotic treatment triggered an increased VLDL/HDL ratio in plasma that may contribute to reducing weight loss observed in treated birds. All metabolic responses co-occurred with significant shift of the microbiota upon infection or antibiotic treatment.

**Conclusion:**

This study indicates that infection and antibiotic treatment trigger dysbiosis that may impact host systemic energy metabolism and cause phenotypic and health modifications.

**Electronic supplementary material:**

The online version of this article (10.1186/s12917-018-1761-0) contains supplementary material, which is available to authorized users.

## Background

Gut microbiota (GM) composition is known to strongly influence host health by a wide range of mechanisms ranging from control of immune functions [1], metabolic homeostasis [2, 3] and drug metabolism [4]. Even if generally stable within a species, the GM composition is strongly impacted by environmental exposure (nutrition, xenobiotics and infection) and any modification of this ecosystem can affect host health by altering the symbiotic relationship existing between the host and its gut microbes [5]. For instance, presence of an opportunistic pathogen in the digestive tract can be asymptomatic but also induce severe health damage. Furthermore, infection is generally associated with bacterial dysbiosis in the digestive track [6], but the impact of such modification on the host metabolism and development of symptom such as weight loss is still poorly understood. Improvement of symptoms is generally observed post antibiotic treatment due to reduction in the number of pathogenic bacteria and the decline of the sequelae of their infection. However, antibiotic use is also associated with a reduction of GM diversity that has been linked to further host metabolic weakening [7].

Avian intestinal Spirochaetosis (AIS) is caused by the colonization of bird’s lower digestive tract by the pathogen *Brachyspira pilosicoli* (phylum Spirochaetes; class Spirochaetes; order Spirochaetales; family Bracyspiraceae) [8, 9]. The bacterium attaches to the cell wall and may trigger diarrhea associated with decreased growth rate and egg production [10]. The most common treatment used to tackle infection is Tiamulin™, an antibiotic of the pleuromutilin family that inhibits protein synthesis by binding to the 50S region of the ribosome [11, 12]. Only a few studies have evaluated its efficiency in chickens despite its intensive use to treat avian flocks in industry [13, 14]. To date this disease and its treatment have been little studied and remain poorly understood. Indeed, the cause for symptoms such as weight loss and decreased egg production are still unclear. In a recent study [15], the efficiency of three doses of Tiamulin™ to treat laying hens orally challenged with *B. pilosicoli* B2904 was evaluated and revealed that infection was associated with decreased growth rate and that birds treated with Tiamulin™ recovered from infection regardless of the dose used while weight maintenance was only observed in response to the two highest doses. Furthermore, Tiamulin™ reduced significantly other infection-associated symptoms as well as systemic spread of *B. pilosicoli*. Nevertheless, three weeks after antibiotic treatment ended, colonization of the digestive track by the pathogen was still observed. Thus, we concluded that this study represented an interesting infection model to understand host systemic metabolic and gut microbiota response to colonization of the digestive tract by a pathogen. In addition, the experimental design allows a longitudinal exploration of the effects of antibiotic treatment on a superorganism (i.e. the host and its gut microbiota). In this paper, we present results obtained following the analysis of biopsy and biofluid samples collected during the previous study [15]. To evaluate host systemic metabolic response to infection and antibiotic treatment we used ^1^H-NMR-based metabolomics that allows a non-targeted evaluation of metabolic fluctuations occurring in biological systems. As the gut microbiota are inextricably linked to host’s metabolic responses, its composition in response to infection and treatment was monitored using 16S rRNA gene sequencing (16S NGS). Both analyses provided new insights into the impact of infection and antibiotic treatment on host health, explaining physiological response to both bacterial and chemical exposure.

## Results

### Infection and antibiotic treatment impact growth and egg production

**Fig. 1:**
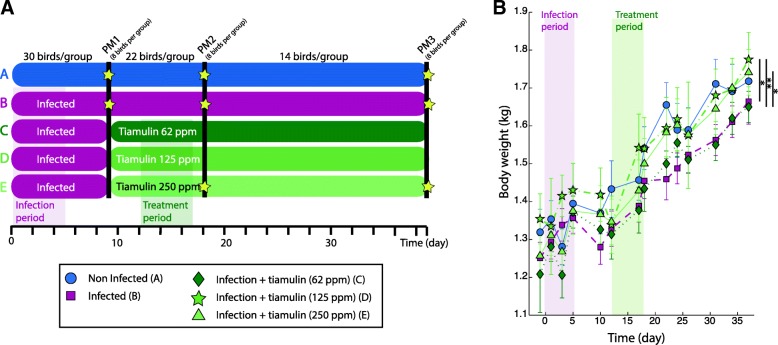
Experimental plan (**a**) and birds body weight (**b**)

Impact of infection and egg production was monitored along the study in all groups (A, control; B infected only; C-E, infected and treated with Tiamulin™ from lowest to highest dose). Infection by *B. pilosicoli* resulted in a significantly decreased growth rate (Fig. 1b) but by the end of the study, chickens from group B (Infected) weighed less than birds from group A (Control) but this result was not significant. The two highest Tiamulin™ doses (group D and E) were able to maintain chicken’s growth since birds from these two groups presented higher weights than those in the control group by the end of the study and that this was significantly higher to the infected group (*p*-value < 0.05). However, animals treated with the lowest dose (Group C), displayed an average weight at the end of the study that was similar to the one of the infected group (B) and significantly different to the other three (A, D and E). This suggested that only higher doses of antibiotics were associated with maintain growth rate during the infection.

### Infection induces systemic metabolic response of the host

**Fig. 2:**
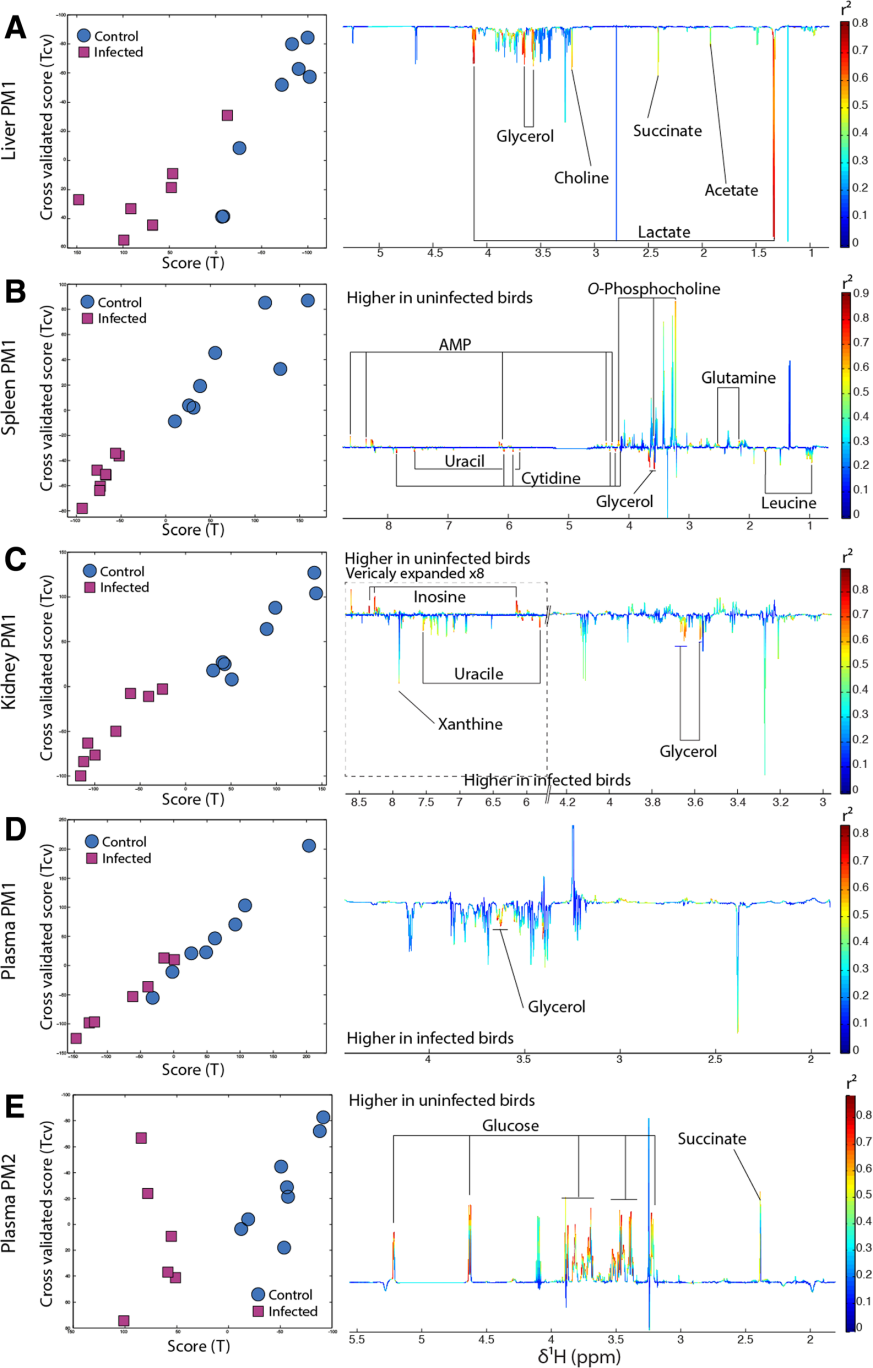
*B. pilosicoli* infection is associated with major systemic metabolism modifications. **a** Scores (right panel) and loadings (left panel) plots of the O-PLS-DA model calculated using 1D-NMR spectra of birds’ liver at PM1 as a matrix of independent variables and infection as a predictor infected birds (red square) and uninfected birds (blue circle). Loading plots shape represent the mean standard deviation of all NMR spectra acquired for the given model and multiplied by the O-PLS DA model weight that allow to visualize if metabolites are positively associated with infection (pointing downwards) or negatively (pointing upwards). The color scale represent the level of correlation between each data point and infection. **b** Same for the spleen. **c** same for the kidney. **d** same for the plasma. **e** same for the plasma at T1

Systemic metabolic response to infection by *B. pilosicoli* was observed directly after the end of the challenge period (day 6). O-PLS-DA revealed that infection was associated with a modification of kidney, liver, spleen and plasma metabolomes (Fig. 2a, b, c and d). Livers of infected birds were richer in glycerol, lactate, choline, succinate and acetate (Fig. 2a). In the spleen, infection resulted in decreased *O*-phosphocholine, glutamine and AMP and increased glycerol, uracil, cytidine and leucine (Fig. 2b). In kidney, infection induced an increase in glycerol, uracil and xanthine contents, concomitant with a decrease in inosine (Fig. 2c). Increased betaine and glycerol were also associated with infection in plasma (Fig. 2d). After infection (PM1), the content of the colon of infected birds was richer in polysaccharides and amino acids (Additional file 1). Two weeks after the end of the infection period (PM2), kidney, liver and spleen of infected but not treated birds had recovered their metabolic homeostasis (there were no more detectable metabolic differences between the control and any other group), which indicates that no metabolic variations were observed in response to infection. However, the glucose level dropped in plasma of infected birds (Fig. 2e) by approximately 50%.

In the ileum, colon, caeca and pancreas no significant metabolic variation in response to infection was observed throughout the study.

By the end of the study it was not possible to differentiate metabolically infected from uninfected birds.

### Tiamulin™ treatment attenuates the metabolic response to infection

We next investigated whether Tiamulin™ modulates the metabolic responses of the host to infection. At PM2, a higher plasma level of betaine was observed in response to infection (*p*-value< 0.01 -Additional file 2). However, birds infected and treated with Tiamulin™ presented similar plasma level of betaine as the controls although this response was not dose dependent.

In the previous section, it was described that infection induced a drop in glucose in chicken plasma (*p*-value< 0.05 –Wilcoxon test-) at PM2 that was partially alleviated by Tiamulin™ treatment. This was not fully corrected by Tiamulin™ treatment since the decrease in plasma glucose level was still lower than in the control group (*p*-value< 0.05). Interestingly, plasma glucose levels were inversely proportional to treatment dose (Additional file 3).

### Tiamulin™ treatment induces a major shift in lipid metabolism

**Fig. 3:**
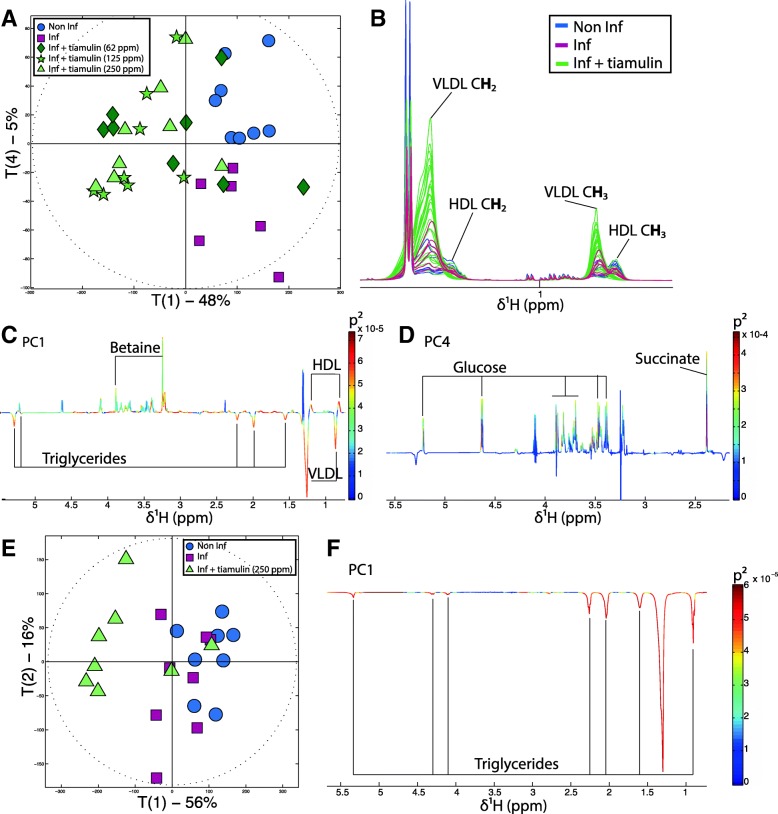
Tiamulin induces plasmatic metabolic variations. **a** PCA score plot on the first (T1 48%) and the fourth (T4 5%) principal component derived from the model calculated using the 1d-NMR spectra of birds’ plasma at PM2. **b** Color-coded plot of the plasma 1D-NMR spectra of control birds (blue), infected and non-treated birds (pink) and treated birds (green). **c** Plot of the principal component 1 (PC1) loadings, molecules pointing up positively correlated with PC1, molecules pointing down negatively correlated with PC1. **d** Plot of the principal component 4 (PC4) loadings, molecules pointing up positively correlated with PC4, molecule pointing down negatively correlated with PC4. **e** PCA scores plot derived from the model calculated using the ^1^H HR-MAS NMR spectra acquired from intact liver biopsies. **f** Plot of the loadings of principal component 1 (PC1) of the PCA model presented in E

The PCA score plot displaying the general impact of treatment on plasma metabolic profiles at PM2 (Fig. 3a) revealed a clear separation between the scores of the birds treated with Tiamulin™ and those un-treated on principal component 1 (PC1). Indeed, scores of antibiotic treated birds occupied a distinct metabolic space from control and infected but untreated birds. Plasma metabolic profiles of chickens treated with Tiamulin™ were characterized by increased very low-density lipoprotein (VLDL) and decreased high-density lipoprotein (HDL) levels (Fig. 3a, b and c). A linear regression of the metabolic profiles against the dose of antibiotics revealed that the effect on lipoproteins was dose dependent (Additional file 4).

Since the liver is the central regulating organ for cholesterol and lipid metabolism, metabolic profiles of intact liver biopsies were generated using HR-MAS ^1^H-NMR spectroscopy. This analysis revealed that the liver of birds treated with Tiamulin™ were richer in lipoproteins than non-treated birds (Fig. 3c and f) suggesting that the liver secreted more VLDL and confirmed the impact of Tiamulin™ on central lipid and cholesterol metabolism.

### Tiamulin™ accelerates post-pubertal metabolic shift

**Fig. 4:**
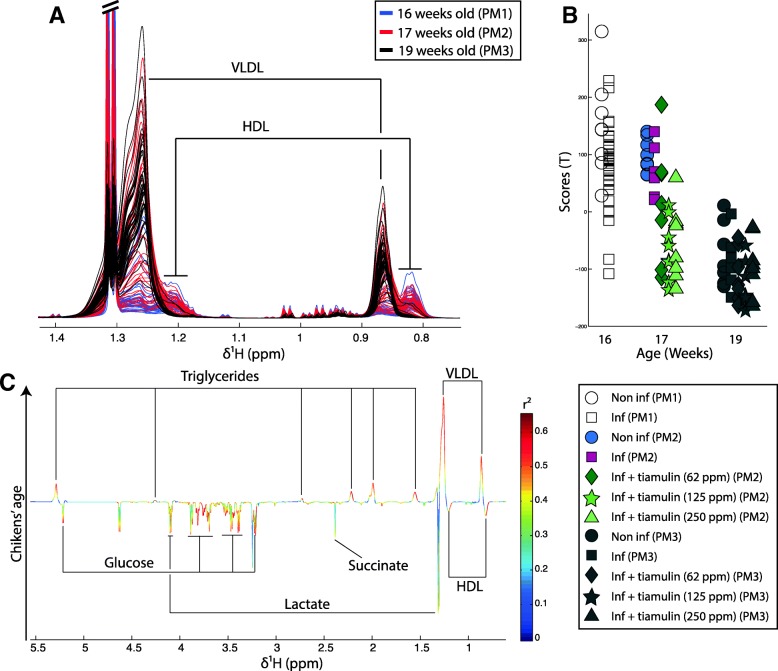
Age is related to increased VLDL and decrease HDL and glucose level. **a** Color-coded plot of the plasma 1D-NMR spectra of 16 weeks old birds (blue), 17 weeks old birds (red) and 19 weeks old birds (black). **b** Plot of the scores of the O-PLS regression model calculated using ^1^H-NMR spectra of birds at all time point as a matrix of independent variables and the birds’ age as a predictor. **c** O-PLS regression coefficient plot related to the birds age

When looking at the impact of Tiamulin™ on chicken plasma metabolic profiles from the overall study (all groups PM1, 2 and 3), it appeared that age was also a strong source of metabolic variation (Fig. 4). Indeed, the linear regression calculated on the plasma metabolic profiles using age as a predictor returned a good model as indicated by strong parameters (R^2^Y = 0.52, Q^2^Y = 0.51 and *p*-value = 0.002). Bird age was associated with decreased HDL, glucose, succinate and lactate level, while VLDL levels increased (Fig. 4). Analysis of the scores (Fig. 4b) revealed that Tiamulin™ treated birds were metabolically similar to post-pubertal birds (PM3 = 19 weeks) at PM2 (= 17 weeks) and that to the contrary, untreated birds had similar metabolic profiles as birds from the pre-pubertal group (PM1 = 16 weeks).

### Infection and Tiamulin™ shifted caecal microbiota composition

**Fig. 5:**
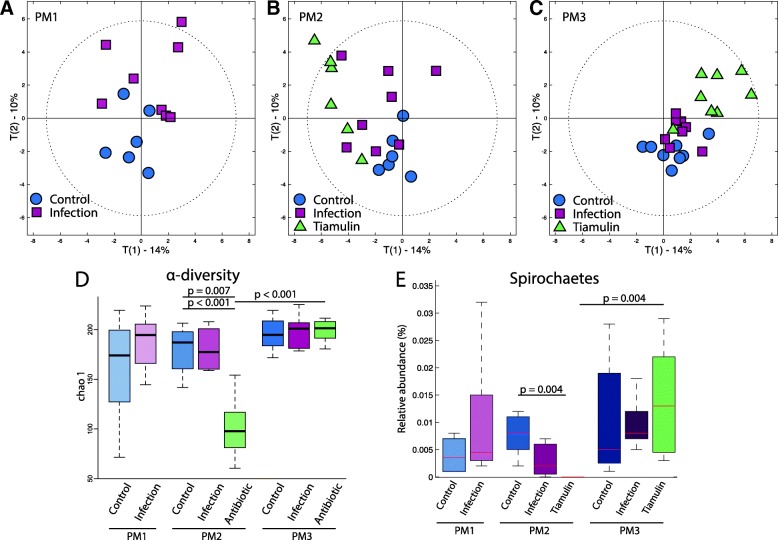
Tiamulin treatment enhances a profound alteration of gut microbial diversity and population. **a** PCA score plots calculated using the bacterial relative percentage of abundance at family level for all birds but displaying only the scores (*n* = 8) of control (blue circle) and infected birds (pink square) post infection (T0). **b** Same PCA score plot than A but displaying only the scores (*n* = 8) of control (blue circle), infected birds (pink square) and birds treated with highest dose (green triangles) post treatment (T1). **c** Same PCA score plot than A and B but displaying only the scores of control (blue circle), infected birds (pink square) and treated birds (green triangles) three weeks post treatment (T2). **d** Alpha diversity calculated for control, infected and treated birds independently of time. **e** Pie chart presenting the bacterial relative abundance at a phylum level for each group (control, infected and treated) for the three time points chosen in this study

The composition of the caecal microbiota population was evaluated in response to infection and Tiamulin™ treatment using 454 16S pyrosequencing of the V4-V5 hypervariable regions. The caecal microbiota population was stable over time in the control group as shown by the PCA score plots (Fig. 5a to c and Additional file 5).

Infection was associated with a modification of the commensal caecal microbiota in comparison to control (Fig. 5a and b), but community balance was recovered at the end of the study (Fig. 5c). This modification of the caecal microbiota was mainly associated with an increase in Lactobacillales, Burkholderiales and Campylobacterales, these last two orders being members of the Proteobacteria phylum (Additional file 6).

After Tiamulin™ treatment the class of Spirochaetes to which *B. pilosicoli* belongs was no longer detectable by 16S sequencing approaches (Fig. 5e). Yet, this bacterial class reappeared three weeks after the end of Tiamulin™ treatment. Furthermore, it was shown in the previous publication that using more targeted methods, *B. pilosicoli* was detectable in every infected groups throughout the study [15]. Their relative percentage of abundance was higher than in both non-treated groups (up to 25%), which is likely due to the bacteria entering a dormancy state during Tiamulin™ treatment [16].

Using a MANOVA, we observed that α-diversity was significantly associated with age (*p*-value = 0.047) and treatment (*p*-value = 0.001) and the interaction between both factors age*treatment (p-value = 7.1*10^− 5^). As expected, the antibiotic dosing was associated with a strong decrease of the bacterial α-diversity in comparison to the control and the infected groups at PM2 (p-value = 0.007 and 4.8*10^− 4^ respectively, Fig. 5d). However, by the end of the study the α-diversity of the treated group was equivalent to the two other groups at the same time point. Tiamulin™ treatment resulted in a major shift in caecal β-diversity of the microbial community (Fig. 5b and Additional file 5). This was driven by a decreased percentage in the relative abundance of Firmicutes (from 30 to 22%) and an increase of the phylum Bacteroidetes (from 60 to 71%). The Firmicutes/Bacteroidetes ratio was changed from approximately 1:2 to 1:3. Although the microbial diversity evolved over three weeks post Tiamulin™ treatment, it failed to return to the initial composition by the end of the study and continued to harbor a relatively high relative abundance of Proteobacteria (Fig. 5b and c).

## Discussion

Although a few studies have been published [17–20], little is known about the relationship between the resilience of the gut microbiota during intestinal diseases, their recovery after antibiotic treatment and the overall impact on the host metabolism, a knowledge gap that motivated this study. Gastro-intestinal infections often trigger gut microbiota dysbiosis, as does treatment by antibiotics [21, 22]. Gut microbiota composition is recognized for having an important role to play in host growth and severe dysbiosis can be responsible for abnormal development [23, 24]. We determined that the intervention study aiming at an evaluation of the efficacy of Tiamulin™ against AIS [15] would allow us to evaluate whether the decreased growth rate associated with infection was triggered by caecal microbiota dysbiosis. We hypothesized that modifications of the gut microbiota by infection would result in modulations of host metabolic homeostasis that was corrected in this study using Tiamulin™ treatment. Materials used for this paper were sampled from a study that showed significantly decreased growth rates amongst other clinical sequelae of egg laying chickens in response to *B. pilosicoli* infection [15].

The infection resulted in an increase in Proteobacteria of which many are opportunistic pathogens associated with increased risk of diarrhea. Interestingly, Proteobacteria enrichment has been associated with metabolic syndrome [25]. Increase in Proteobacteria has previously been observed in response to Penicillin in mice that was linked to increased body weight, percentage fat mass and diabetes incidence [26].

In the present study, infection and bacterial dysbiosis were concurrent with profound host systemic metabolic changes. The range of tissues affected by infection (liver, spleen, kidney and plasma) indicates a systemic metabolic response of the organism to *B. pilosicoli* colonization and dysbiosis. Interestingly increased glycerol levels were noticeable in all aforementioned compartments. Systemic glycerol increase is a marker of lipolysis in adipose tissues where triglycerides are lysed into free fatty acids and glycerol by lipase enzymes. Glycerol is then released in the general circulation to be used as a glucose precursor in the liver and/or the kidney. This mechanism is generally activated by prolonged low plasma glucose levels. In addition, GI infection can impair glucose absorption due to gut barrier disruption and it thus possible that this phenomenon was also triggered by reduced intestinal glucose uptake. *B. pilosicoli* is known to strongly disrupt the intestinal wall [27], which was further supported by the observation of higher glucose levels in feces of infected birds. The increased polysaccharides in feces may also be associated with the ability of *B. pilosicoli* to degrade mucin [28–30]. Furthermore, the concomitant increase in butyrate and acetate observed during the infection attests higher carbohydrate fermentation and therefore a modification of the GM metabolic activity. Plasma glucose concentration is highly controlled and regulated since its level needs to be maintained to sustain essential functions such as brain and muscular activity. To maintain glucose levels gluconeogenesis from glycerol is activated during fasting, which requires fat storage to release non-esterified fatty acids and glycerol in plasma. This reduction of fat mass is likely to be related to the decreased growth rate observed in chickens colonized by *B. pilosicoli* [15, 31]. However, the drop in plasma glucose level observed at PM2 following recovery of glycerol levels, suggests that this alternative metabolic pathway cannot sustain the energy demand for a long time.

Complete recovery of host metabolic homeostasis in response to infection was reached at the end of the study (PM3). This coincided with a net decrease in percentage of infected birds in all groups [15] and a stabilization of the caecal microbiota. Hence, symptoms and noticeable metabolic responses of the host to infection occurred only when microbiota dysbiosis was observed. Such an observation suggests that both presence of the pathogen and modification of gut microbial community are necessary to trigger host metabolic responses. The idea that the GM might act as a buffer regulating host metabolic response to infection by a pathogen has been partially explored by Khosravi et al. [32], who showed that *Helicobacter pylori* infection triggered a stronger host metabolic response (modification of insulin, ghrelin and leptin levels) in germ free mice than in conventional animals and that infection-induced decreased growth rate was only observed in absence of GM. This tolerance to pathogen has been associated with the training of the immune system. Thus, an infection can be considered as a response to an ecosystem variation rather than to colonization by a single pathogen.

In this set of results, Tiamulin™ was able to reduce infection-induced metabolic response, the betaine increase and glucose drop in plasma. Although a dose response was observed on the level of infection measured by positive swabs [15], this was not true for the plasma betaine levels. It is likely that increased betaine levels in response to infection was related to the central osmoprotectant role of this molecule [33]. Betaine has been used previously as food supplement for chickens due to its ability to protect the gut barrier against pathogens such as *Coccidia* [34]. Indeed, *B. pilosicoli* cell invasion induces swelling and disturbance of the osmotic balance [9, 27]. Increased quantities of betaine (generally coming from kidneys) may therefore be transported from other tissues towards the gut barrier via general circulation explaining its increased level in plasma.

We observed that normal ‘metabolic aging’ (decreased HDL/VLD and glucose plasma levels) occurring at puberty was accelerated by Tiamulin™ treatment. Interestingly, decreased of the HDL/VLDL ratio and glucose levels in the general circulation have been associated with increased levels of steroid hormones and more specifically progesterone [35–37]. Cytochrome P450 3A (CYP3A, an important enzyme family present in the liver involved in drug detoxification) are involved in steroid hormone metabolism (progesterone, estrogen and testosterone). In addition, it was shown in several studies that a decrease in CYP3A activity generally resulted in increased plasma steroid hormone concentrations [38–40]. Antibiotics are active molecules that can interact directly with the host if able to cross the gut barrier. It has been reported that Tiamulin™ interacts with CYP3A, forming a complex that results in the inactivation of the cytochrome in vitro and in vivo [41–44]. Therefore, it is likely that the observed pre-puberty metabolic shift resulted from the interaction of Tiamulin™ with progesterone metabolism. This was further supported by the fact that egg laying onset, which highly depends on steroids metabolism maturation, occurred earlier in the two groups receiving the highest doses of Tiamulin™ [15]. Altogether, this supports the potential interaction of Tiamulin™ with steroid metabolism.

Finally, the lipoprotein shift may also be caused by the gut microbiota modifications due to antibiotic treatment. The host-GM metabolic interplay has been widely investigated. Indeed, many studies have reported that obesity and energy metabolism homeostasis are strongly associated with gut microbiota composition [24, 45–47]. Furthermore, it has been demonstrated that the use of antibiotics before puberty in humans and mice can be associated with increased risk of metabolic disorders due to modulations of the gut microbiota [26, 48, 49]. Interestingly, a low Firmicutes/Bacteroidetes ratio has been reported to be related to a lean phenotype and lower risk of developing disorders characterized by modification of cholesterol metabolism [2, 50, 51]. However, this is contrary to our observations which suggests that the VLDL/HDL modification detected might be driven by the Tiamulin™ itself rather than by microbiota modifications: whether this is a generalizable phenomenon for some or all classes of antibiotic is clearly very worthy of future study. Also we need be mindful that other bacterial changes may be responsible for modification of cholesterol metabolism: indeed some lactic acid bacteria are known to be able to metabolize cholesterol [52] and their use as feed supplement in broiler chickens resulted in decreased plasma cholesterol concentrations [53]. Hence, further experimentation would be needed to tease these aspects apart.

## Conclusion

This work demonstrates that gut microbiota composition can be associated with perturbations of host systemic metabolism that lead towards phenotypical changes. We observed that infection was associated with dysbiosis, decreased nutrient absorption and host energy metabolic disorder that resulted in significantly reduced growth rate. Two systemic biomarkers of infection were identified as glycerol and betaine. Increased systemic glycerol clearly illustrated host metabolic adaptation to intestinal infection directed towards providing sufficient energy supplies for survival. However, impaired weight gain was still observed presumably, as glycerol was likely to be supplied from adipose tissue. In addition, symptoms due to colonization by the pathogen were only observed when associated with gut microbial dysbiosis. This finding strongly supports the potential protective role of the gut microbiota against opportunistic pathogens. This indicates that further studies should be undertaken to understand the ecological context in which a pathogenic bacterium might become harmful for its host. In this study, the antibiotic treatment reduced infection and associated symptoms while modifying cholesterol metabolism. From our results, and previously published work we hypothesized that host metabolic response to antibiotic treatment resulted from a co-occurring modification of the gut microbiota composition and steroids metabolism. These findings suggest that impact of antibiotic consumption on host energy metabolism should be studied as a response of a direct interaction and through mediation of the gut microbiota. Finally, antibiotic triggered a decrease in α-diversity followed by dysbiosis that might lead to higher vulnerability to colonization by pathogen and favor relapse. Therefore, antibiotic treatment coupled to food supplements such as pre/pro/symbiotic should be considered in order to recover a ‘healthier’ gut microbiota post intervention.

## Methods

### Animal study and experimental design

Briefly, 150 16–17 weeks old NovoGen Brown commercial layer hens sourced from a commercial supplier (Tom Barron Ltd., UK) were housed at the APHA (Addlestone, Surrey, UK) according to Home Office guidelines (Home office license -PPL 70/7249-) and all procedures were performed in compliance with the Animals Scientific Procedures Act, 1986. After the study, animals were incinerated onsite to avoid risk of contamination by the pathogen to the environment.

The experimental plan was described previously by Woodward et al. [15] and for clarity is summarized in Fig. 1. The animals were allocated randomly to five groups (*n* = 30) given the following treatments: Group A: Untreated, uninfected controls; Group B: Untreated, infected controls; Group C: Infected + Tiamulin™ at 62.5 ppm; Group D: Infected + Tiamulin™ at 125 ppm; Group E: Infected + Tiamulin™ at 250 ppm.

After crop neutralization, birds were challenged by oral gavage with 1 mL of *B. pilosicoli* B2904 suspension (5 × 10^9^ CFU/ml) for five days every two days [54]. One week after the end of the challenge, group C, D and E received different concentrations of Tiamulin™ in the drinking water for five days. Birds were then observed for three weeks. Feed was un-medicated layer pellets (Dodson and Horrell) and water was provided from the mains supply, birds had access to both ad libitum.

### Sample collection from animal study

Biopsies, plasma and faecal samples were collected during *post-mortem* examination at three time points: the day after the end of the infection process (PM1), the day after the end of the antibiotic treatment (PM2) and at the end of the study (PM3) (Fig. 1a). For each group and time point eight birds were randomly selected and euthanasia was performed by sedation using Rompun/Ketamine mixture as an intramuscular injection followed by an intravenous injection of Pentobarbitone. Blood was sampled first from the heart and serum was frozen at − 80 °C after clot. Tissue biopsy samples (Approximately 1 g for all tissue) and faecal samples collected directly from the intestinal track for both coon and caeca (approx. 1 g) were snap frozen in liquid nitrogen and then stored at − 80 °C for future assessement of their metabolic composition and cecal microbiome profiling.

Data regarding the general impact of infection and Tiamulin™ treatment on birds’ level of infection, growth, health condition (scored via observation of the bird feather and muscular development), egg production, water and food consumption are also explained in the aforementioned article [15] and are not repeated here.

### Sample preparation for NMR

Kidney, pancreas, spleen and liver polar metabolite extraction was done by homogenizing 0.1 g of tissue in 1 mL of 3:1 (*v*/v) methanol/H_2_O solution using a tissue lyser [55]. After centrifugation (10 min at 12000 x g), supernatants were dried in a speed vacuum and resuspended in 600 μL of phosphate buffer (0.2 M) containing 90% of D_2_O and 10% of H_2_O plus 0.01% of sodium 3-(tri-methylsilyl)-propionate-2,3-d4 (TSP used as internal standard). Samples (0.5 mL) were then transferred to 5 mm NMR tubes for acquisition. Plasma samples were mixed to phosphate saline buffer with 90% D2O at a 2:1 (v/v) ratio, 0.5 mL were then transferred to 5 mm NMR tubes. 0.0150 g of liver biopsy were added with phosphate buffer in spinner for solid state NMR spectroscopy.

### NMR spectroscopy

For tissues ^1^H-NMR spectra were acquired on a 700 MHz Bruker Advance Spectrometer using a standard noesypr1D pulse program with water presaturation (relaxation delay of 2 s and 100 ms of mixing time). Plasma 1D NMR spectra were acquired using a Carr-Purcell-Meiboom-Gill (CPMG) pulse. Liver biopsies were acquired on 500 MHz Bruker Advance Spectrometer using a ^1^H HR MAS probe. Spectra were acquired using a standard noesypr1D pulse as well as CPMG. For all matrixes, 2D NMR experiments were run on selected samples to help metabolites identification as well as a previously published chicken metabolic atlas [55]. Spectra were acquired with using 256 scans with 16 dummy scans (DS). All spectra were recorded as 64 k data points (15 ppm).

### DNA extraction methods for 16S based population studies

DNA from faecal samples were extracted using PowerSoil® DNA Isolation Kit (*MO BIO Laboratories, Inc)* purchased by Qiagen. To ensure DNA samples quality, PCR of the universal V4-V5 region of the 16S rRNA was performed post extraction (cycling conditions: 94 °C for 3 min; 30 cycles of 94 °C for 30 s, 55 °C for 45 s, 72 °C for 1 min; followed by 72 °C for 8 min) and concentration was assessed using a Nano drop. PCR primers were the following:

- U515F: 5’-GTGYCAGCMGCCGCGGTA
- U927R: 5’-CCCGYCAATTCMTTTRAGT

### Next generation 16S sequencing

Aliquots of extracted DNA were amplified with universal primers for the V4 and V5 regions of the 16S rRNA gene. The primers U515F (5’-GTGYCAGCMGCCGCGGTA) and U927R (5’-CCCGYCAATTCMTTTRAGT) were designed to permit amplification of both bacterial and archaeal ribosomal gene regions [56]. Forward fusion primers consisted of the GS FLX Titanium primer A and the library key (5’-CCATCTCATCCCTGCGTGTCTCCGACTCAG) together with one of a suite of eight 10 base multiplex identifiers (MID) (Roche Diagnostics Ltd., UK). Reverse fusion primers included the GS FLX Titanium primer B and the library key (5’-CCTATCCCCTGTGTGCCTTGGCAGTCTCAG). Amplification was performed with FastStart HiFi Polymerase (Roche Diagnostics Ltd., UK) using the following cycling conditions: 94 °C for 3 min; 25 cycles of 94 °C for 30 s, 55 °C for 45 s, 72 °C for 1 min; followed by 72 °C for 8 min. Amplicons were purified using Ampure XP magnetic beads (Beckman Coulter) and the concentration of each sample was measured using the fluorescence-based Picogreen assay (Invitrogen). Concentrations were normalized before pooling samples in batches of up to 16, each of which would be subsequently identified by its unique MID. Pooled samples were then subjected to unidirectional sequencing from the forward primer on the 454 GS FLX Titanium platform according to the manufacturer’s instructions (Roche Diagnostics).

The Ampliconnoise pipeline [57] was used to split the dataset into separate files for each sample according to the MID adaptors used, and then to remove pyrosequencing errors, PCR errors and chimeric sequences. Only sequences over 400 bases in length were retained for further analysis. The processed sequences were then classified using the pick open reference OTUs process implemented in QIIME v1.9.1 (Caporaso, et al. 2010) against the Greengenes 16S rRNA gene database (http://greengenes.secondgenome.com/downloads/). The resulting distribution of OTUs across the multiple samples was further analyzed using QIIME v1.9.1. to summarize the distributions and explore alpha and beta diversity [58].

### Statistical analysis

For metabonomics analysis, after applying exponential window with line broadening of 0.3 Hz and Fourier transformation, spectra were individually phased and base line corrected on the software MestReNova (Mestrelab Research v.8.1.2). Spectra were then imported in Matlab (the Mathwork ® v2013a) where they were calibrated on TSP (δ 0.00) for all tissue extract, lactate (δ 1.33) for plasma and the H^1^ proton of α-glucose (δ 5.23) for liver biopsy. Spectra were normalized for each matrix individually using a probabilistic quotient method [59]. Metabolic variation between samples was evaluated using principal component analysis (PCA). This step was also used to remove potential outliers that were considered as such if acquisition had failed to provide a spectrum comparable to the other samples of the same set. When group clusters of interest were spotted, orthogonal projection to latent structure discriminant analysis (O-PLS DA) was used to evaluate metabolic variation between groups using NMR spectra as a matrix of independent variables and infection or treatment as a prediction vector. Algorithm for regression models were provided by Korrigan Sciences Ltd.

The Wilcoxon test was used to evaluate significance in variations between groups in regard to weigh and α-diversity using R. A MANOVA test was also performed to identify the impact of time, infection and treatment on α-diversity on R (model <− aov(α-diversity ~ time*infection*treatment). Finally, due to sequencing depth and method, we decided to pursue the microbial community analysis at the family level and not at lower taxonomical level. Statistical analyses were performed on zero inflated log transformed relative abundance. A total of 54 families were detected but only 40 were present in at least 25% of the samples. Βeta diversity at the family level was performed by calculating the Euclidian distance between individual.

## Additional files

Additional file 1: Infection modifies GM metabolic activity and polysaccharide intestinal lumen content. (A) OPLS-DA scores against cross-validated scores calculated using faecal water spectra of group A and B at PM2 and infection as a predictor. (B) Loading plot associated to the OPLS-DA model described in A. (EPS 14763 kb)

Additional file 2: Plasma level of betaine at PM2 for all groups, A: control, B: Infected, C: infected and treated (62 ppm), D: infected and treated (125 ppm), E: infected and treated (250 ppm). * pv < 0.05; ** pv < 0.01. (EPS 614 kb)

Additional file 3: Glucose plasma level at PM2 for all groups, A: control, B: Infected, C: infected and treated (62 ppm), D: infected and treated (125 ppm), E: infected and treated (250 ppm). * pv < 0.05; ** pv < 0.01. (EPS 601 kb)

Additional file 4: Linear plasma response to Tiamulin treatment dose. (A) Plot of the scores against the cross-validated scores of the O-PLS regression model calculated using ^1^H-NMR spectra of birds at PM2 as a matrix of independent variables and Tiamulin doses as a predictor. Model parameters: R^2^Y = 0.48, Q^2^Y = 0.43 and *p*-value = 0.01 (EPS 13970 kb)

Additional file 5: Relative abundance in percentage of the Spirochaetes OTU for each treatment group along the study. (EPS 948 kb)

Additional file 6: Loadings associated to the PCA scores plot in Fig. 5 calculating using the relative abundance of OTUs for all samples as a matrix of independent variable. A, loadings of PC1. B, Loadings of PC2. (EPS 2121 kb)

Additional file 7: **Table S7**. OTU raw read counts table. (CSV 3322 kb)

Additional file 8: **Table S8**. Sample key and metadata for Additional file 7: Table S7. (CSV 2 kb)

Additional file 9: Supplementary Material S9. ARRIVE cheklist. (PDF 1067 kb)

## Abbreviations

HDL: High density lipoprotein
NMR: Nuclear magnetic resonance
O-PLS DA: Orthogonal projection to latent structure discriminant analysis
PCA: Principal component analysis
PM: Post mortem
VLDL: Very low density lipoprotein
